# Computational Network Inference for Bacterial Interactomics

**DOI:** 10.1128/msystems.01456-21

**Published:** 2022-03-30

**Authors:** Katherine James, Jose Muñoz-Muñoz

**Affiliations:** a Northumbria University, Faculty of Health and Life Sciences, Department of Applied Sciences, Newcastle upon Tyne, United Kingdom; University of California San Diego

**Keywords:** interactome, interologs, data integration, cellular network analysis, systems biology

## Abstract

Since the large-scale experimental characterization of protein–protein interactions (PPIs) is not possible for all species, several computational PPI prediction methods have been developed that harness existing data from other species. While PPI network prediction has been extensively used in eukaryotes, microbial network inference has lagged behind. However, bacterial interactomes can be built using the same principles and techniques; in fact, several methods are better suited to bacterial genomes. These predicted networks allow systems-level analyses in species that lack experimental interaction data. This review describes the current network inference and analysis techniques and summarizes the use of computationally-predicted microbial interactomes to date.

## INTRODUCTION

The representation of biological data as a network, in which nodes represent biological entities and connecting edges represent associations between them, is both visually and computationally tractable (1). Many graph-theoretic tools have been developed to reveal these networks’ properties and identify their properties of biological relevance (2–5). Network approaches have been used to investigate maps of protein interactions, termed “interactomes” (6), in several eukaryotic model species using data produced using a number of high-throughput experimental techniques (7–15).

The interactomes of most organisms are largely uncharacterized due to time and cost constraints, and the lack of culture-based technologies for many species. In microbes, the first large-scale bacterial interactome produced was for the gastric pathogen Helicobacter pylori using the yeast two-hybrid approach (16), which identifies protein–protein interaction between bait and prey proteins via activation of a reporter gene following reconstitution of its transcription factor. This network was later expanded to cover ∼70% of the proteome (17). Escherichia coli has by far the most experimental interaction data, including binary protein–protein interaction (PPI) data produced using yeast two-hybrid (18) and protein complex data from affinity purification (19–21). Several large transcriptional, metabolic, and regulatory data sets are also available for this species (22–26). Experimental interaction data sets have been produced for a number of other medically-important pathogenic microbes, including Campylobacter jejuni (27), Mycobacterium tuberculosis (28), Mycoplasma pneumoniae (29), Streptococcus pneumoniae (30), and Treponema pallidum (31). Interaction data have also been produced for the plant symbiote Mesorhizobium loti (32), the model bacterial species *Synechocystis* sp. PCC6803 (33), and Bacillus subtilis (34, 35). In addition, a complete complex interactome network has been described for the gut microbiome community describing the microbe–microbe and host–microbe talk elucidating the role of the gut microbiota in health and disease (36).

## COMPUTATIONAL INTERACTOME PREDICTION

Several computational PPI prediction methods have been developed that harness existing data (37–39). These methods can be loosely grouped as similarity-based, genome context, evolutionary, and machine learning.

## SIMILARITY-BASED

**FIG 1:**
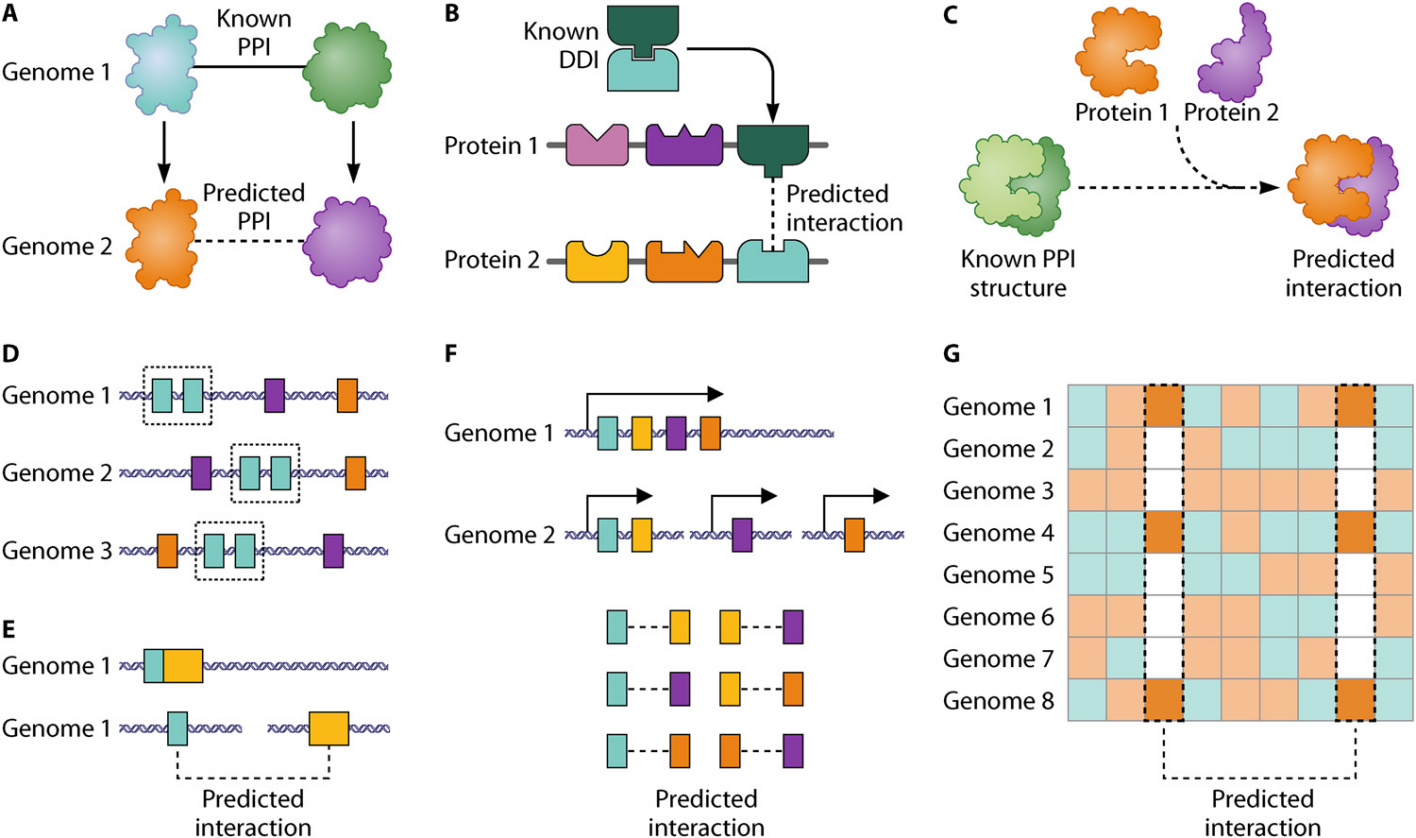
Protein–protein interaction (PPI) inference methods. (A) Interologs: where experimentally confirmed interaction partners in one species have similarity to proteins in another species, an interaction can be predicted. (B) Domain–domain interactions (DDIs): the presence of a pair of domains with a known interaction can be predictive of PPI in other proteins containing those domains. (C) Structural interaction: protein structures can be mapped to the structure of interacting proteins to infer PPI. (D) Gene neighborhood: conservation of protein pairs’ (green) proximity in multiple genomes can be predictive of interaction between the pair. (E) Gene fusion: proteins that are fused in one species (yellow and green) have a potential PPI in species in which they are separate proteins. (F) Gene cluster: transcription from an operon in one species indicates functional relation and often PPI in another. Here, an operon of four proteins in one species is predictive of six interactions in another. (G) Phylogenetic profile: protein pairs that interact often have a similar pattern of conservation in multiple genomes (green, presence; orange, absence).

PPIs and their network topology are conserved (40–45). In particular, highly-connected network hub proteins tend to be essential, have slower evolutionary rates, and conserved interactions (3, 46), even between eukaryotes and prokaryotes (47) Protein–protein interactions, termed “interologs,” can consequently be transferred between species (48–52) (Fig. 1A). Conservation has also been observed in regulatory (49, 53), functional (54), and co-expression networks (55–59), allowing transfer of interactions in the same way. Since protein domains are vital to function, the presence of pairs of domains can be predictive of PPI (Fig. 1B), even in proteins with relatively low sequence-similarity (60–62). Domain–domain interaction (DDIs) and interologs are often used in combination to improve network inference (63–67). Similarity of the three-dimensional protein structures can also be used to predict their interactions (Fig. 1C) (68–70). The main prediction methods involve homology modeling (mapping to the known structure of a homologous protein) (71, 72), threading (mapping to known structure of nonhomologous proteins) (73, 74), and docking (predicting the 3D orientation of two interacting proteins) (75–77).

## GENOME CONTEXT

Genome context prediction methods compare the location of pairs of genes across multiple genomes (40, 78, 79). “Gene neighborhood” infers interaction based on the assumption that interacting proteins are more closely located on the genome. As an example, in *Bacteroides* spp., genes for SusC-SusD pairs are close in the genome of the bacteria. By comparison of multiple genomes, conservation of pairs’ proximity can be identified (Fig. 1D) (80, 81). Although conservation of gene order has been observed in mammals (82), neighborhood-based prediction is most accurate in bacterial genomes (83). “Gene fusion” events can indicate protein interactions since proteins that are fused in one species have a potential functional link in other species in which they are separate proteins (Fig. 1E) (84–86). The “gene cluster” prediction method assumes that transcription from an operon in one species indicates functional relation and often interaction in another (Fig. 1F) (79, 81).

## EVOLUTIONARY

Interacting protein pairs tend to evolve at the same rate (46, 87), and so the distribution of protein pairs will therefore be co-conserved if they interact (78, 88–91). The phylogenetic profile method (Fig. 1G) infers interactions when two genes have a similar pattern of conservation (presence/absence) in multiple genomes (92, 93). MirrorTree and ContextMirror algorithms extend this method beyond binary similarity to globally assess the phylogenies (94, 95). Finally, *in silico* two-hybrid (i2H) method identifies interacting pairs from correlated mutations in multiple sequence alignments, since mutation in interacting proteins co-evolve (96). Several other extensions to these methods have been proposed (97–99).

## MACHINE LEARNING

Machine learning can be used to infer PPI by training a classifier on positive (interacting) and negative (noninteracting) pairs of proteins (100). Many data types can be included in the training set including sequence features, co-citation, protein annotation, phylogenetics, expression data, and physiochemical properties (101–109), and multiple data types produce better accuracy than a single input (110, 111). The resulting PPI networks often have confidence scores for putative interactions that allow thresholding and the use of network analysis algorithms that utilize these scores (112). Microbial PPI prediction has been carried out using several algorithms including random forests (113–117), support vector machines (118–120), and Bayesian classifiers (121–123). A related method is probabilistic functional integrated networks (PFINs), which combine multiple data types in a probabilistic framework to produce a network of confidence-weighted interactions (124–126).

## NETWORK VALIDATION

Once a predicted interactome has been built, it is essential to evaluate how accurately it represents real cellular biology and assess the level of false interactions that may be present. Evaluation of the quality of a predicted network is difficult due to the level of noise in the underlying data: often several validation methods are required. Small-scale experimental validation can be used on a subset of PPIs to give a level of confidence in the predicted network (20, 64, 122, 127, 128). However, experimental validation is only possible for a small number of interactions. Expression data sets can be used to assess PPIs since interacting proteins are likely to have correlated expression (64, 119, 128–131), and evidence of support for predicted PPIs can be found in other experimental data sets or from text-mined small-scale studies (122, 132–134).

Domain data can be used to assess networks predicted using other input data types (133). Similarly, interaction predictions from other methodologies can be assessed using phylogenetics (119, 122, 129, 131). Hub proteins in predicted networks often correspond to hubs in other species (135). Clustering the network can be used to assess how well the network represents known protein complexes (136). Comparison of the predicted network with random networks can also be used to assess its biological relevance (64, 137).

Protein functional annotations provide useful validation tools as interacting proteins tend to have shared function (64, 119, 128, 131, 132, 138, 139), shared cellular localization (128, 132, 138), and related phenotype (119, 131, 133). Protein functional prediction can provide an objective method of network evaluation by testing its ability to predict the known annotations, for example by a leave-one-out or partitioned cross-validation (140–143). Data partitioning can also be used in the training/testing phase of machine learning to provide a measure of network accuracy (134).

## NETWORK ANALYSIS

Several network parameters can be used to reveal aspects of network topology and identify key proteins (144). The *degree* of a protein is its number of interactions; proteins with a high degree are considered hubs and tend to be essential and conserved (46, 145, 146), and are often targets for pathogens (147, 148). Identification of hubs in predicted networks can highlight important proteins for further study (129). The *degree distribution* of a network, *p*(*k*), is the probability a selected protein has *k* links (149). This distribution reflects the organization of cellular processes (150), with many low degree proteins and a small number of hubs giving a *scale-free* distribution (4, 149, 151). This topology makes networks resistant to random perturbation (5, 151) and has been found in several biological networks in a number of model species (17, 152–154), although some do not have this topology (27, 155).

Biological networks are considered *small world* since they have small diameters (longest shortest path between two proteins) and small characteristic path lengths (average shortest path) relative to equivalently-sized random networks (156–158). The proteins of these networks are arranged in locally-dense regions interconnected by a small number of interactions, and, like scale-free networks, this topology is resistant to perturbation (5).

Several network measures assess the importance of proteins and interactions in networks’ information flow to identify bottlenecks. For instance, *betweenness centrality* (159) measures the proportion of shortest paths passing through a protein/interaction. Proteins with high betweenness centrality and low degree often link network modules (160). Betweenness centrality can aid the identification of key proteins within a predicted network (116, 147).

Biological networks tend to have a hierarchical structure of modules within modules (4, 161). Dense network regions are believed to relate to the functional units of the cell (151, 162–165). Partitioning or clustering large networks can reveal the underlying mechanisms of cellular biology and assign protein function (137, 166, 167). Module detection can use additional data, for instance gene expression data (168), functional annotations (169), or domain profiles (170).

Predicted networks can be used to directly annotate proteins with function (171). Network-based annotation transfers known annotations between pairs of directly connected proteins (172, 173), between proteins with shared interaction partners (174), or more globally using network topology (175). Interaction confidence weights, such as those produced by machine learning algorithms, are particularly useful for annotation transfer (173). Annotation and other data can also be used to create process/condition-specific subnetworks (176, 177).

## NETWORK COMPARISON

Network comparison can reveal underlying network properties, detect noise, predict missing data, and reveal conserved interactions (41, 178–180). Heuristics, such as global properties and local motifs, are commonly used for comparison (181–183), although some nonheuristics have been developed (184). At the simplest, level biological networks can be compared to network models (185) in which interactions are randomized, while topological characteristics, such as degree distribution and diameter, are preserved, to produce a network profile; similarity of profile indicates underlying similarities of the networks. Networks can be compared directly by comparison of topological properties; however, two networks with similar topology can be vastly different (186). An alternative approach is to analyze the distribution of network motifs (187).

Network alignment produces a more accurate method of comparison (41, 188). Within-species alignment is relatively straightforward since proteins can be merged based on identity (151, 188) and overlap between networks can be used to identify true interactions (189). At a more complex level, networks can be compared across multiple species, either locally by aligning small conserved regions or globally across the network structures (41, 178, 182, 190–194). Alignment complexity increases with the size of the networks and with the number of networks to be aligned (191).

## MICROBIAL INTERACTOME NETWORKS

While prokaryotes have far outpaced eukaryotes in the production of sequence data, the opposite is true for interaction data. Largescale experimental data are only available in a few species, and small-scale studies require considerable curation to analyze as a whole (195, 196). However, several studies have produced a number of predicted interactomes (Table 1), providing insights into several aspects of microbial biology. Additionally, the STRING database and server contains functional interaction data, including co-citation, co-expression and gene neighborhood, for multiple microbial species (197). STRING data have been used as the initial data source for network studies in several species (115, 178, 198–204).

**TABLE 1:** Predicted bacterial interactomes

Species	Methodology^*a*^	Proteins	Interactions	Source
Actinobacillus pleuropneumoniae	ORTH	533	2,737	242
Agrobacterium tumefaciens	ORTH	296	690	142
Bacillus anthracis	ORTH	264	732	142
Bacillus licheniformis	ORTH, DDI, GE	2,448	15,864	139
Bacillus subtilis	ORTH	247	707	142
Brucella melitensis	ORTH	238	652	142
Brucella suis	ORTH	225	611	142
Campylobacter jejuni	DDIs	-	-	207
	ORTH	334	1,028	142
Clostridium difficile	RF: STRING, GO	-	955	115
Corynebacterium pseudotuberculosis *^b^*	ORTH, STRING	-	15,495	200
Escherichia coli	DDIs	-	1,280	207
	ORTH	400	1,473	142
	SVM: GC, CL, PP	3,798	78,122	134
	ML: GC, PP, MT, CM, IH	4,150	1,847,729	121
	EXP, GC	4,146	80,370	20
	PFIN: EXP, DDI, GE, CC, GC, PP	4,099	95,520	124
	PP	1,479	1,618	205
Helicobacter pylori	ORTH	771	5,647	142
Klebsiella pneumoniae	PFIN: ORTH, GC, DDI, PP, GE, CC	4,674	160,450	125
Listeria monocytogenes	ORTH	176	485	142
Methanobrevibacter ruminantium	STRING, GC, MET	637	2,194	202
Methanothermobacter thermautotrophicus	STRING, GC, MET	256	2450	201
Mycobacterium tuberculosis	STRING	3,925	29,664	198
	ORTH	738	5,639	213
	RF: STRING, GO	-	1,854	115
	ORTH, SVM: SEQ	3,465	46,119	119
	STRING	144	587	199
	PP	1,020	911	205
Pseudomonas aeruginosa	ORTH	333	903	142
	RF: GE, CL, GN, DDI, SEQ, FUN	4,181	54,107	116
	PFIN: CC, DDI, GC, GE, ORTH, PP	5,456	203,118	126
Pseudomonas putida	ORTH, DDI	3,254	82,019	210
Salmonella enterica ^*b*^	ORTH, EXP, STRUCT, MET, TF	30,870	81,514	220
Salmonella typhimurium	ORTH	332	1,359	142
Shigella flexneri	ORTH	383	4,548	142
*Synechocystis* PCC6803	DDI, STRUC, STRING	2,930	109,532	204
	ORTH, DDI, GO	998	8,783	51
	NB: ORTH, DDIs, GC	3,231	4,715	122
Vibrio cholerae	ORTH	275	1.021	142
Vibrio parahaemolyticus	ORTH	365	1,520	142
Vibrio vulnificus	ORTH	372	1,557	142
Yersinia pestis	ORTH	352	1,100	142
Xanthomonas oryzae	ORTH, DDI	1,988	36,886	260

a CC: co-citation; GE, gene expression; CL, cellular localization; CM, context mirror; DDI, domain–domain interaction; EXP, experimental; FUN, functional interaction; GC, genome context; GO, gene ontology; IH, *in silico* two hybrid; MET, metabolic interactions; ML, machine learning; MT, mirror tree; NB, Naïve Bayes; ORTH, orthology (interologs); PP, phylogenetic profile; PFIN, probabilistic functional integrated network; SEQ, sequence properties; STRING, https://string-db.org; STRUC, structural interactions; SVM, support vector machine; TF, transcription factor interactions.

b Combined for multiple strains.

## CELLULAR BIOLOGY AND PROTEIN FUNCTION

In E. coli, an interactome study combined experimental data with genome context predictions to assign functions to proteins, including several involved in cell envelope biogenesis (20). A phylogenetic profile-based network was later produced, which contained previously uncharacterized components of several complexes, including the ribosome (205). Comparison of these networks (20, 205) using edge propagation, demonstrated that both identify complexes overlapping functional modules (206). A support vector machine-derived E. coli network was shown to be scale-free and had good overlap with experimental data (134). The EcID database incorporated genome context and phylogenetic evidence into a Bayesian classifier to predict protein function, in particular linking *yeaG* and *yeaH* to nitrogen metabolism (121). EcoliNet is a probabilistic functional integrated network for E. coli comprising ∼99% of the genome and has successfully predicted knockout phenotypes (124). The interacting domain profile pair method, IDPP, was evaluated on E. coli before producing a network for C. jejuni from H. pylori interaction data (207, 208). IDPP was shown to successfully predict interactions in the target species that were not found in the source species.

Incorporating expression data, interologs and DDIs, Bacillus licheniformis proteins were assigned to complexes and putative functions (139). A probabilistic network for Klebsiella pneumoniae, derived from multiple data types, was used to identify antibiotic resistance genes (125). Wuchty and colleagues expanded experimental networks of Streptococcus pneumoniae and H. pylori using interolog data to improve their functional predictive power (30, 209). Interologs and DDIs were combined with experimental data to study the metabolic modules of Pseudomonas putida (210). In the related species P. aeruginosa, probabilistic functional network integration identified novel virulence and antibiotic resistance genes (126).

SynechoNET is a predicted interactome for *Synechocystis*, focused on membrane biology (204), while InteroPORC predicted an interactome for this species comprising 28% of the genome (51). Later Naïve Bayesian network classification was applied to protein functional prediction and modular analysis in *Synechocystis* (122). Interactome prediction has also been widely used to study Mycobacterium tuberculosis due to unavailability of accurate *in vitro* methods in this bacterium (211, 212). Several predicted interactomes have been created to study network properties (119), evolution (205), protein function (115), virulence (213), and drug resistance (198) in this species. Finally, interolog networks for 22 bacterial species (the largest of which are included in Table 1) were produced by McDermott and colleagues and used to predict functions for a large number of unannotated bacterial proteins (142) demonstrating the potential of large-scale network studies to enhance our understanding of bacterial cellular physiology.

Computational prediction has also been used to understand the interplay between bacteriophages and bacteria (214). Phylogenetic profiles using genomic/metagenomic data have identified host–virus and virus–virus relationships (215–218). Leite and colleagues used interactions, DDIs, and sequence properties to compare machine learning frameworks, concluding that predictive power will improve as input data increases (114). More recently, a Markov random field framework of virus–host and virus–virus similarity measures has been developed (219).

## INTERACTOME EVOLUTION

Comparison of networks for different species can reveal insights into interactome evolution. Zitnik and colleagues used STRING data to create networks for 1,539 bacteria (178); comparison with those of eukaryotes revealed that interactomes have evolved to become more robust, and that bacterial interactome robustness is associated with more complex environments. Using a binary interaction data set for Treponema pallidum, interolog networks were created for 372 other genomes, ∼28% of which were estimated to be true interactions (31). This study also revealed that bacterial proteins have higher degrees than eukaryotic proteins. These networks also revealed a central role for cell motility proteins in bacterial interactomes.

By comparing a Methanobrevibacter ruminantium network with those of *Methano-sarcina acetivorans*, Methanosarcina barkeri and Methanococcus maripaludis biosynthetic subsystems involved in survival in the rumen were identified (202). By comparing the Methanothermobacter thermautotrophicus metabolic interactome with those of metal-loving bacteria, separate evolution of niche-specific cellular functions was revealed (201). A comparison of 10 strains of Salmonella enterica identified distinct transcription factor targets conferring adaptation to gastrointestinal and extra-intestinal environments (220). Similarly, comparison of host–pathogen interactomes between two strains of Burkholderia pseudomallei revealed several interactions unique to the virulent strain and highlighted the potential roles of chaperon and drug/carbohydrate binding proteins during infection (221).

## PATHOGEN–HOST INTERACTIONS

Interactome prediction can identify cross talk between pathogen and host (222, 223). A DDI-based network suggested that human–M. tuberculosis PPIs tend to have more domains than intraspecies interactions (138), and this trend was later observed in an interolog-based mapping study, which also revealed that hub proteins of intraspecies networks tend to be involved in host–pathogen PPI (148). Using a random forest framework, the cancer pathway was involved in M. tuberculosis infection (117), while a DDI network implicated several PPIs involving heat shock, redox proteins (224). Finally, a combination of interolog and DDI mapping associated several genes of the host immune responses to M. tuberculosis infection (65).

In Fusobacterium nucleatum, a host-pathogen network implicated the Fap2 adhesin as a virulence protein (225). Comparison of machine learning classifiers for Bacillus anthracis–humans PPI prediction, suggested neural networks outperform SVMs (120); the resulting interactions revealed involvement of apoptosis and immune regulation pathways in infection. The predicted networks between humans and B. anthracis, Francisella tularensis, and Yersinia pestis indicated that hubs and bottlenecks of the intraspecies networks tend to interact (147, 226). Thirteen membrane proteins of Leptospira interrogans were predicted to be involved in cellular disruption during infection, four of which were common between strains (203). Coelho and colleagues produced a human–microbial PPI network of the oral cavity using a Bayeisan classifier, which revealed Rothia mucilaginosa, Leptotrichia buccalis, and Actinomyces odontolyticus as having the most interactions with human proteins (123).

In the plant pathogen Ralstonia solanacearum, interolog network analysis identified interactions between its transportation proteins and core proteins of the *A. thaliana* interactome (227). The response to metal ions was linked to the host defense response during Pseudomonas syringae infection of *A. thaliana* (228). Defense response proteins were also found to be enriched in random forest-derived networks between *A. thaliana* and the pathogens P. syringae, *Hpaloperonospora arabidopsis*, and *Golovinomyces orontii* (113). Interolog and DDI mapping has also been used to study plant–bacterial symbiosis, suggesting a role of host 14-3-3 and heat shock proteins in the relationship between Bradyrhizobium diazoefficiens and Glycine max (229).

## VIRUSES

Computational prediction has been applied to viral species including HIV (230–232), hepatitis C virus (233), human papillomaviruses (234), and Ebola (235). In particular, the recent pandemic has highlighted the importance of understanding viral–host interaction, and having resources available to rapidly respond to new viral threats. Two studies have compared PPI prediction to emerging data from Sars-CoV-2: the first used interolog mapping, DDIs, and machine learning to link ACE2 and DPP4 to spike protein binding (236); the second used an ensemble machine learning algorithm based on experimental data and sequence features to predict >1,000 potential human protein targets (237).

## DRUG TARGETS

Interactome networks can aid in the identification of potential drug targets by revealing essential pathogen interactions (238). A M. tuberculosis–human interactome was shown to be enriched in predicted drug targets (239), and several studies have used computational prediction to identify putative drug targets (199, 213) and to understand the mechanisms of drug resistance in this important pathogen (198, 240). In *P. aeroginisa* a random forest predicted network was used to prioritize drug targets based on their essentiality and topological importance (116). Using interolog mapping, 12 putative drug targets were identified in methicillin-resistant Staphylococcus aureus, including a histone deacetylase (241). A predicted Corynebacterium pseudotuberculosis interactome was used to identify 41 essential proteins as candidates for infection diagnosis in livestock, highlighting the tryptophan biosynthesis pathway as a potential drug target (200). The network also revealed that this species may use multiple iron acquisition strategies in low iron environments. In the swine pathogen Actinobacillus pleuropneumoniae, nine drug target candidates were identified using interolog mapping (242). Interolog analysis has also been applied to the gut microbiome to identify target species driving metabolic change during disease (243).

## CONCLUSIONS AND FUTURE PERSPECTIVES

Although PPI network prediction has been extensively used in eukaryotes, microbial network inference can be achieved using the same principles and analysis techniques. Bacterial interactomes share common hierarchical properties, such as modularity and robustness (244). Many of the caveats to interactome prediction in eukaryotes, such as evolutionary distance, unequal conservation, and physiological context (245–249) are mitigated in prokaryotes due to their smaller genomes and single-celled nature. Several prediction methods, in particular gene neighborhood and gene cluster, are more suited to microbial than eukaryotic use (79, 83), and phylogenetic profiles are powerful predictors, particularly when including inputs from the three domains of life (250).

Interolog and DDI mapping can only detect interactions within conserved areas of the genome (48), and these methods rely on the quality of the underlying interaction data; stochastic activation of reporters can give false positives and low sensitivity leads to false negatives (251, 252), and different methods have their own strengths (253–256). In eukaryotes, poor overlap has been observed between data sets of different types, and between those of the same type (10, 189). Comparison of experimental data of C. jejuni, H. pylori, and E. coli suggests that these data sets have significant levels of overlap and similar rates of false results (27). Meta-interactome analysis can be used to identify broadly-conserved biological systems, although levels of conservation remain low due to lack of experimental interactome coverage in many species (257).

Current experimental interactome data are incomplete and biased toward well-studied proteins and species (178). Using a combination of computational methods (51, 119, 139, 201, 210), and experimental data if available (20, 124, 220), gives a more complete predicted interactome, reduces some biases, and strengthens the evidence of true interactions. Integration of diverse data types is particularly effective when using a probabilistic (124–126) or machine learning (115, 116, 121, 134) framework, allowing thresholding of interaction confidence scores and therefore reduction of noise.

Filling in the gaps in bacterial interactomes is vital to our understanding of their biology, and computational prediction can help to pin down these areas and target further analyses by identifying areas of interest and providing putative protein functions. Microbial comparative interactomics is now possible on a large scale; for instance, the >1,500 predicted networks produced by Zitnik and colleagues revealed the evolutionary rewiring of interactomes through time (178). While there are parts of some interactomes that cannot currently be predicted due to the complexity of the protein locations and the lack of the accurate annotations in the genome of new bacterial isolates, interactome accuracy will improve as coverage of diverse species increases, providing insights in several areas of biology, in particular the identification of PPIs for antibacterial discovery (258), understanding of pathogenicity through host cell rewiring (256), and in engineering of synthetic cellular systems (259).
